# Case series of four re-infections with a SARS-CoV-2 B.1.351 variant, Luxembourg, February 2021

**DOI:** 10.2807/1560-7917.ES.2021.26.18.2100423

**Published:** 2021-05-06

**Authors:** Thérèse Staub, Vic Arendt, Esther Calvo Lasso de la Vega, Pierre Braquet, Christian Michaux, Michel Kohnen, Chantal Tsobo, Tamir Abdelrahman, Anke Wienecke-Baldacchino, Jean-Hugues Francois

**Affiliations:** 1 Infectious Diseases Department, Hospital Center Luxembourg (CHL), Luxembourg; 2 Microbiology Department, Department (CHL), Luxembourg; 3 Microbiology department, National Public Health Laboratory (LNS), Luxembourg

**Keywords:** COVID-19, outbreak, variant, re-infection, PCR, healthcare workers, SARS-CoV-2

## Abstract

We describe four SARS-CoV-2 re-infections with a B.1.351 variant in 2021, in healthcare workers (HCWs) previously infected in 2020, before detection of this variant in Europe. Cases live in France, near the border with Luxembourg, where variants B.1.351 and B.1.1.7 circulated. All work in the same hospital unit where a cluster of COVID 19 with B1.351 variant occurred, affecting patients and HCWs. Before the cluster onset, HCWs used surgical masks, as per recommendations. After cluster onset, HCWs used FFP2 masks.

Since the description of the first cases of coronavirus disease (COVID-19) caused by severe acute respiratory syndrome coronavirus 2 (SARS-CoV-2) in Wuhan, China, in late December 2019, more than 141 million cases and 3 million deaths have been reported worldwide [1]. The duration of protective immunity against the virus is still unknown but is thought to be several months, estimated by the persistence of neutralising antibodies [2,3]. Some cases of re-infections with SARS-CoV-2 have been documented but are rare [4-6]. The SARS-CoV-2 B.1.351 variant was first identified in December 2020 in South Africa and is one of the recently identified variants of concern (VOC) [7].

We describe a series of four cases of re-infection with the B1.351 variant in Luxembourg, in 2021, in healthcare workers (HCWs) who had been previously been infected with SARS-CoV-2 in 2020, before the B1.351 variant was first detected in Europe.

## Case series

Case 1 is a male HCW in his mid-20s living in France, who had travelled to the United Arab Emirates in November 2020. Before flying back to France, a real-time reverse transcriptase (RT) polymerase chain reaction (PCR) was performed in a nasopharyngeal swab that was positive for SARS-CoV-2. His only symptoms were loss of smell and taste over a period of 2 weeks. He has no known underlying conditions. Serology from a blood sample drawn on 7 January 2021 showed 74.2 U/mL of anti-spike IgG. In February 2021, when a cluster of COVID-19 occurred in the hospital unit where he worked, he developed a cough but no fever. On 16 February, while he was still asymptomatic, a screening RT-PCR from a nasopharyngeal swab was positive for SARS-CoV-2 and consecutive virus sequencing confirmed the B1.351 variant.

Case 2 is a female HCW in her mid-20s also living in France. She had a first episode of COVID-19 in April 2020 with fever, headache, chills, diarrhoea, loss of taste and smell, confirmed by a positive PCR in a nasal swab. She has no known underlying conditions. A serology performed on 31 December 2020 showed anti-spike antibodies at 25 U/ml. In February 2021, when there was a COVID-19 cluster in the hospital unit where she worked, she developed fever, chills, and headache, and a RT-PCR from a nasopharyngeal swab was positive on 12 February, the day of symptom onset. The sequencing of the virus confirmed the B1.351 variant.

Case 3 is a female HCW in her late 30s living in France. She did not have a symptomatic first episode of COVID-19, however, a serology performed on 26 January 2021 showed 68.9 U/mL anti-spike IgG. She has no known underlying conditions. She developed chills, myalgia, and headache on 12 February 2021, when there was a COVID-19 cluster in the hospital unit where she worked. A RT-PCR from a nasopharyngeal swab was positive 3 days later, on 15 February. The sequencing confirmed the B1.351 variant.

Case 4 is a female HCW in her late 20s living in France. In November 2020, she had a symptomatic SARS-CoV-2 infection with fever, muscle pain, headache, loss of taste and smell. She has no known underlying conditions. A RT-PCR from a nasopharyngeal swab was positive for SARS-CoV-2 on 18 November and viral sequencing could unfortunately not be performed retrospectively because of low viral load in the sample. Anti-spike IgG determined in a serum sample on 31 January 2021 was 131 U/mL. In February, during the COVID-19 cluster in the hospital where she worked, she presented with muscle pain and cough. A RT-PCR from a nasopharyngeal swab was positive on 16 February, the day of symptom onset, and viral sequencing confirmed the B1.351 variant.

The four HCWs live in an area in France, in Moselle near the border with Luxembourg, from where circulation of B1.351 and B.1.7. 7 was reported [8]. All worked in the same hospital unit where a cluster of COVID-19 infections caused by a SARS-CoV-2 B1.351 variant occurred; this cluster affected 21 patients and 19 HCWs between 12 and 26 February 2021. As per existing recommendations for the non-COVID-19 units, the HCWs followed general hygiene precautions and wore surgical masks before the cluster onset. After the beginning of the cluster, the use of filtering facepiece (FFP2) masks was recommended.

## Laboratory investigations

We used a multiplex real-time RT-PCR, Taqpath Covid-19 CE-IVD RT-PCR (Thermofisher scientific, Waltham , United States (US)), targeting and amplifying genomic sequences of the spike (S) protein, nucleocapsid (N) protein and the open reading frame 1ab (ORF1ab) genes, to detect SARS-CoV-2. Immunoglobulin detection of total antibodies (isotypes G, M and A) against epitopes of the receptor binding domain in the spike S1 subunit, was performed using the SARS CoV-2 electro-chemiluminescence assay (ECLIA) Elecsys Anti-SARS-CoV-2 S on a cobas e 801 (Roche diagnostics, Basel, Switzerland).

For whole genome sequencing, we extracted viral RNA from nasopharyngeal swabs. Using the nucleic acid extracts, we generated the whole genome of SARS-CoV-2 by amplicon sequencing adapting the ARTIC Network protocol for a paired-end 150bp strategy (dx.doi.org/10.17504/protocols.io.bbmuik6w). The library preparation was carried out with the Nextera Flex DNA kit (Illumina, San Diego, US)) following the manufacturer’s instructions, and adapted for an amplicon length-dependent clean-up step. The samples were processed for sequencing on an Illumina MiniSeq platform. We created the consensus sequence applying a reference-based mapping with bowtie2 (version 2.3.4.1) [9] using the RefSeq-sequence of Wuhan-1 sequence (GenBank Accession ID NC_045512_2). Downstream analysis was carried out for the generated consensus sequence if it met two criteria: (i) a minimum call depth of 20X and (ii) reference sequence coverage of 90%. We assigned clade and lineage by using the assign clade script of next strain [10] and Pangolin (version 2.0.5), respectively (2020).

Sequences were deposited in the Global Initiative on Sharing All Influenza Data (GISAID). (GISAID) EpiCoV database. All three sequences with coverage above 90% are identical when using pairwise genetic distance (GISAID numbers: EPI_ISL_1384095, EPI_ISL_1384130, EPI_ISL_1384139). The fourth sequence is identical in all regions that are covered and comparable to the other sequences, however, due to low coverage, no conclusion could be made on the full genome. The low coverage was due to low viral load (cycle threshold value: 35) which was at the limit of sequencing method, and for any sample above that threshold yielding low coverage in sequencing, data are excluded from downstream analysis.

### Ethical statement

Ethical approval was not required for this study as all four HCWs gave their consent for the publication.

## Discussion

All four HCWs presented in this series had a first episode of SARS-CoV-2 infection before December 2020. At that time, the B1.351 variant was not circulating in Europe. For three of them, a PCR test was performed during the first episode and all had anti-spike IgG serum concentration of minimum 25 U/mL before their re-infection in 2021, confirming their first infection episode.

In the case of SARS-CoV-2 antibodies, the most important neutralising potential is ascribed to antibodies against the S protein, given its role in binding to and fusing to the targeted host cells, and its exposure to the immune system [3]. Because of its binding and fusing functions the S protein has been chosen as the target for vaccines. Owed to natural constraints in production, currently authorised COVID-19 vaccines are mostly based on the well characterised historical SARS-CoV-2 variants. Titres of anti-S antibodies seem to be higher on average in vaccinated subjects (2 weeks after receiving the second dose of the mRNA-based vaccines) than following natural infection [11].

To our knowledge, only one other case of re-infection with the B1.351 variant has been described in the literature thus far. The patient had severe illness requiring intensive care and mechanical ventilation [12]. The four HCWs reported here were all young without comorbidities, they all recovered and their re-infections were not severe. Moreover, the re-infection was not more severe than the first episode for each of them.

At the onset of the COVID-19 pandemic there were no known clinically relevant differences between variants. Since the end of 2020, however, several VOC have emerged, most notable for their mutations in parts of the S-protein, potentially changing their binding and fusing efficiency. The SARS-CoV-2 B.1.1.7 (also referred to as 501Y.V1 or VOC202012/01) variant was identified for the first time in late 2020 in the United Kingdom, while the B.1.351 (also referred to as 501.V2 variant) was first identified in South Africa and the P.1 variant in Brazil. Higher transmissibility of the VOCs has been observed and led to dominant circulation since the emergence in different regions globally [13]. These variants all carry the D614G mutation in the spike protein, this mutation leads to higher transmissibility and make these VOC predominant worldwide [7]. Furthermore, because of their S-protein genetic shift, some of the VOCs may either be unaffected by neutralising antibodies either induced by vaccination or by previous infections of other variants, or may require higher antibody titres than can be reached by natural infections or even vaccination [13,14].

The Elecsys anti-spike kit used in our investigation has been compared with the first World Health Organization standard provided by the British National Institute for Biological Standards and Controls [15], by its manufacturer who reports a correlation of 1 Roche Arbitrary Unit/mL to 0.972 binding antibody unit (BAU)/mL (IC95%: 0.956–0.987) and thus a rough agreement of both units. Furthermore, the manufacturer compared their kit to a commercially available CE-IVD, enzyme-linked immunosorbent assay (ELISA)-based surrogate neutralisation assay (cPass, Genscript, Nanjing, China). They established that 15 U/mL seemed to be a suitable threshold of agreement to presence of neutralising antibodies (defined as ≥ 20% of inhibition for this kit) and they reported a complete inhibition (80–100%) starting titres at 200 and above [16].

No agreed threshold of protection has been established to this date and the main purpose of performing serology in a routine hospital setting remains the demonstration of earlier contact with the virus itself or, more recently, with a spike-based vaccine. Given that reinfection in these four cases with B.1.351 occurred at previous titres ranging from 25.2 to 131.0 U/mL (or BAU/mL), this seems to be a prudent approach to rather consider the presence of antibodies for confirming a past infection than to determine a protection. Two different phylogenetic strains by whole genome sequencing must be detected to confirm re-infection. Even though we were not able to compare the sequences from the first infections to the second ones, the fact that the B1.351 variant had not been detected in Europe at the time of the first infection episodes is strong evidence for the re-infections.

## Conclusions

Re-infection is possible with the B1.351 variant in people who had a first asymptomatic or symptomatic infection with one of the historical SARS-CoV-2 strains between 3 to 12 months earlier. Anti-spike antibodies may perhaps not protect against a re-infection with the B1.351 variant. The number of re-infections described here was limited and re-infections should be further studied in larger populations to determine their frequency. The re-infections we observed were in young HCWs without underlying conditions and disease was not severe.
